# 570 Efficacy of Nebulized Heparin in Inhalation Injury: 12 Years Single Center Experience

**DOI:** 10.1093/jbcr/iraf019.199

**Published:** 2025-04-01

**Authors:** Adalah Yahia, Sarah Jaszcz, Marco R Scipione, Lester Laddaran, Michael White

**Affiliations:** Detroit Receiving Hospital Burn Center; Detroit Medical Center; DMC Detroit Receiving Hospital; Detroit Medical Center; Michael & Marian Ilitch Department of Surgery, Wayne State University School of Medicine, Detroit Medical Center

## Abstract

**Introduction:**

Inhalation injury is a significant contributor to morbidity and mortality in burn patients. Nebulized heparin is hypothesized to reduce fibrin casts and lung obstruction, potentially improving clinical outcomes such as time on mechanical ventilation, mortality, and pneumonia rates. Current evidence is limited to small retrospective studies, with conflicting results regarding the efficacy of nebulized heparin.

**Methods:**

This retrospective cohort study reviews 12 years of experience at a single Burn Center treating inhalation injuries. Patients who received nebulized heparin (10,000 units every four hours) from January 2016 to December 2022 were compared to those who received only standard care (albuterol, ipratropium, or n-acetylcysteine) from January 2010 to December 2015. Adult patients requiring mechanical ventilation due to inhalation injury were included. The primary endpoint was 28-day ventilator-free days. Secondary endpoints included ICU and hospital length of stay, 7-day extubation rate, mortality, pneumonia incidence and timing, and bleeding events.

**Results:**

A total of 162 patients met inclusion criteria: 73 in the heparin group and 89 in the non-heparin group. Baseline characteristics were largely similar across groups, though patients in the heparin group were more likely to receive other nebulized agents (n-acetylcysteine, albuterol, and ipratropium). No significant differences were observed between the groups in terms of 28-day ventilator-free days (11.1 vs. 16.5 days, p = 0.289), 7-day extubation rates (30% vs. 28%, p = 0.775), mortality (18% vs. 21%, p = 0.573), pneumonia incidence (67% vs. 64%, p = 0.682), time to pneumonia (5.8 vs. 4.6 days, p = 0.692), or bleeding rates (68% vs. 55%, p = 0.081).

**Conclusions:**

This study demonstrates that nebulized heparin does not reduce mechanical ventilation duration, mortality, or pneumonia rates in patients with inhalation injury. However, nebulized heparin appears to be safe for use in this population. Future prospective studies are needed to further assess whether nebulized heparin offers clinical benefits in terms of ventilator duration, pneumonia, or mortality, as suggested by prior retrospective studies.

**Applicability of Research to Practice:**

Nebulized heparin is commonly used in the treatment of smoke inhalation injuries. As the largest retrospective analysis to date, this study did not show significant improvements in ventilator duration, mortality, or pneumonia rates but confirmed the safety of nebulized heparin. Further prospective studies are needed to determine if it offers clinical benefit.

**Funding for the Study:**

N/A

